# Mouse innate resistance to *Neospora caninum* infection is driven by early production of IFNγ by NK cells in response to parasite ligands

**DOI:** 10.1128/msphere.00255-24

**Published:** 2024-10-24

**Authors:** R. S. Coombs, A. E. Overacre-Delgoffe, A. Bhattacharjee, T. W. Hand, J. P. Boyle

**Affiliations:** 1 Department of Biological Sciences, University of Pittsburgh, Pittsburgh, Pennsylvania, USA; 2 Department of Immunology, University of Pittsburgh, Pittsburgh, Pennsylvania, USA; Cleveland State University, Cleveland, Ohio, USA

**Keywords:** *Neospora caninum*, *Toxoplasma gondii*, innate immune response, interferon γ, natural killer cells

## Abstract

*Toxoplasma gondii* is capable of being transmitted by nearly all warm-blooded animals, and rodents are a major source of parasite dissemination, yet mechanisms driving its broad host range are poorly understood. Although a phylogenetically close relative of *T. gondii*, *Neospora caninum* differs from *T. gondii* in that it does not infect mice and only infects a small number of ruminant and canine species. We recently showed that *T. gondii* and *N. caninum* grow similarly in mice during the first 24 h post-infection, but only *N. caninum* induces an IFNγ-driven response within hours that controls the infection. The goal of the present study was to understand the cellular basis of this rapid response to *N. caninum*. To do this, we compared immune cell populations at the site of infection 4 h after *T. gondii* or *N. caninum* infection in mice. We found that both parasites induced similar frequencies of peritoneal monocytes, while macrophages and dendritic cell populations were not increased compared to uninfected mice. Through a series of knockout mouse experiments, we show that B, T, and NKT cells are not required for immediate IFNγ production and ultimate control of *N. caninum* infection, suggesting that natural killer (NK) cells are the primary inducers of immediate IFNγ in response to *N. caninum. N. caninum* infections exhibited significantly more IFNγ^+^ NK cells in the peritoneum compared with *T. gondii*-infected and uninfected mice. Finally, we demonstrate that differences in early IFNγ production during *N. caninum* and *T. gondii* infections in mice are at least partly due to differences in soluble antigen(s) produced by tachyzoites.

**IMPORTANCE:**

Pathogen differences in host range are poorly understood at the molecular level even though even closely related pathogen species can have dramatically distinct host ranges. Here, we study two related parasite species that have a dramatic difference in their ability to infect mice. Here, we show that soluble proteins from these species determine one driver of this difference: induction of interferon gamma by cells of the innate immune system.

## INTRODUCTION

*Neospora caninum* and *Toxoplasma gondii* are closely related apicomplexan parasites with substantial genetic similarity but distinct host ranges. *T. gondii* is capable of infecting all warm-blooded animals and causes disease in numerous species including humans. *N. caninum* infects and causes disease in ruminant and canine species and is specifically problematic to cattle and dairy industries worldwide. A large body of work has identified natural killer (NK) cells as IFNγ producers during acute *T. gondii* infections and describe an IL12-dependent mechanism for NK IFNγ production (1–5). Although fewer studies have addressed NK cell activity during *N. caninum* mouse infections, NK cells were identified as important early responders during bovine neosporosis and are detected within days of infection in circulation (6). Importantly, bovine NK cells can be directly stimulated by *N. caninum* to produce IFNγ and exert cytotoxicity against *N. caninum*-infected cells (7).

Dendritic cells (DCs) and macrophages also play a crucial role in controlling acute *T. gondii* infections as major sources of IL12 (1, 8). Additionally, monocytes are required for host survival to *T. gondii* and are key to expanding macrophages and DC populations during infection (9). Inflammatory monocytes migrate to the site of infection and differentiate into macrophages and DCs, which then rapidly respond to infection through phagocytosis, antigen presentation, and production of IL12 and other cytokines. Although there are tissue-resident populations of both cell types, it is primarily inflammatory (infiltrating) monocyte-derived cells that produce IL12 at the site of *T. gondii* infection (10). NK-derived IFNγ is critical for monocyte differentiation at the site of infection and IFNγ which in turn increases local production of IL12 by monocyte-derived DCs (10, 11). Recent work identified bone marrow NK cells producing IFNγ prior to the onset of systemic inflammation during acute *T. gondii* infections. Importantly, they showed that NK-derived-IFNγ in the bone marrow phenotypically alters monocytes before they egress, ultimately changing their function in target tissues (12). These examples highlight how local infections alter monocyte cell fate during hematopoiesis, and how IL12 and IFNγ production can impact innate cell populations prior to systemic inflammatory responses to *T. gondii*.

Despite robust myeloid IL12 production and NK cell IFNγ production in response to *T. gondii* infection, *T. gondii* is a highly successful parasite of mice and other rodents in the wild, capable of infecting and being transmitted by multiple rodent species. In contrast, *N. caninum* is not known to be an effective parasite of rodents, and our prior work demonstrated a clear link between the timing of IL12 and IFNγ production and the differences in rodent susceptibility to *T. gondii* and *N. caninum* (13). Comparisons between *T. gondii* and *N. caninum* revealed that a robust proinflammatory cytokine profile is produced as early as 4 h after *N. caninum* infection, including significant levels of IFNγ. Although *T. gondii* infections induce IFNγ, it is usually not detected until 2–5 days after infection, long after *N. caninum* proliferation has been controlled (13). The cellular source of IFNγ during *N. caninum* infection is not known. While NK cells are the major producers of IFNγ during *T. gondii* infection, other innate immune cells have also been implicated (14). IFNγ-producing cells during *T. gondii* infections include innate lymphoid cells (ILCs) including NK cells, neutrophils (15, 16), iNKT cells, CD8 and CD4 T cells (17), and γδ T cells (18). The goal of the present study was to (i) determine if *N. caninum* infection recruits distinct cell populations compared to *T. gondii*, (ii) to better understand which cell types may be required for early control and IFNγ production in the first hours of *N. caninum* infection, and (iii) identify potential sources of the species-specific IFNγ stimulation profiles.

## MATERIALS AND METHODS

### Parasite strains, maintenance, and infection

Parasite strains *N. caninum* Nc1:LucDsRed and Nc1:Nc1 WT (Nc:Nc1), as well as *T. gondii* TgS1T, TgS1T:Luc:DsRed, and TgMe49 were maintained in human foreskin fibroblasts as previously described (13). Cells and parasites were maintained in Dulbecco’s modified Eagle medium (DMEM) supplemented with 10% fetal bovine serum, penicillin (100 U/mL), streptomycin (100 µg/mL), and L-glutamine (2 mM; cDMEM) and grown in humidified, 5% CO_2_ incubators at 37 degrees. To obtain parasites for infections, cells were passed through 25- and 27-gauge needles, pelleted by centrifugation, and resuspended in 3 mL phosphate-buffered saline (PBS). Parasites were quantified using a hemocytometer, and concentrations were adjusted by dilution or concentrated with centrifugation.

### Animals

Experiments were performed with 6–8-week-old female mice obtained from Jackson Laboratories. The following mice were ordered from the Jackson Laboratory: C.129S7(B6)-Rag1tm1Mom/J (Stock No: 003145), C;129S4-Rag2tm1.1Flv Il2rgtm1.1Flv/J (Stock No: 014593), C.129S2-Cd1tm1Gru/J (Stock No: 003814), Balb/C (Stock number: 000651), and C57BL6 (Stock number: 000664).

### Sample preparation for flow cytometry

Balb/C mice (6–8 weeks old) were infected with 10^6^ tachyzoites of *N. caninum* Nc1 strain or *T. gondii* S1T strain. For flow cytometry experiments, mice were infected with parasite strains that did not express mCherry or luciferase to increase the number of markers that we could use. Mice were sacrificed 4 h after infection, and peritoneal and spleen samples were collected from infected mice and additionally collected from uninfected (naive) mice for comparisons. Peritoneal exudate cells (PECs) were collected by peritoneal lavage with 5–10 mL of Ice cold RPMI media containing 3% fetal bovine serum (FBS), 1:1,000 50 mM beta mercaptoethanol, 1:100 penicillin (100 U/mL), streptomycin (100 µg/mL), and 1:50 HEPES. PECs were then washed and incubated with the selected antibody cocktails. For spleen samples, the spleens were harvested from the infected and naïve mice described above. Spleens were suspended in the media described above and kept on ice. Spleen cells were suspended using a 70 µM filter and washed using centrifugation and media. Red blood cells were lysed using 1 mL ammonium-chloride-potassium lysis buffer and washed again. The step was repeated once if necessary, and then cells were incubated with the appropriate antibody cocktail.

### Flow cytometry

Splenocytes and PECs were stained using the following directly labeled antibodies and dilutions: Cd45.2 clone 104 (1:200), CD11b clone M1/70 (1:200), CD11c clone HL3 (1:200), MHC II clone M5/114.15.2 (1:200), Ly6G clone 1A8 (1:200), Ly6C clone HK1.4, DX5 (1:200), CD3 clone 17A2 (1:200), TCRβ clone H57-597 (1:100), CD90.2 clone 30-H12 (1:800), CD4 clone RM4-5 (1:200), CD8b clone H35-17.2 (1:200), CD8a clone 53-6.7 (1:200), IFNγ clone XMG1.2 (1:200), NK1.1 clone PK136 (1:100), and γδTCR clone GL3 (1:800). Dead cells were excluded using LIVE/DEAD fixable stain (ThermoFisher). All stains were performed in media containing anti-CD16/32 blocking antibody (clone 93). For intracellular staining for cytokines, cells were incubated with 5 µg/mL Brefeldin-A for 3.5 h at 37°C, following which they were fixed and permeabilized using the FoxP3/Transcription factor staining buffer set according to the manufacturer’s directions prior to IFNγ detection using intracellular cytokine staining.

### Bioluminescent imaging, serum collection, and cytokine analysis

As previously described (13), we infected 6–8-week-old female mice (Balb/C, and knockout lines outlined above; Jackson Laboratories) via intraperitoneal (IP) injection of 10^6^ luciferase-expressing (Luc) *N. caninum* (Nc1:LucDsRed) or *T. gondii* (TgS1T:Luc:DsRed) parasites in 200 µL sterile PBS. Bioluminescent imaging was obtained by injecting mice with 3 mg D-luciferin in 200 µL sterile PBS, and images were taken using the IVIS Lumina II imaging system (Xenogen Corporation). Images were analyzed using Living Image software to calculate the total flux (photons/s) across the entire body of the mouse. Serum samples were obtained via submandibular venipuncture to quantify serum cytokine levels via Luminex processed and analyzed at the University of Pittsburgh Medical Center CFP Luminex Core Laboratory. Samples were analyzed using the Procarta-Plex TH1-TH2 mouse panel to quantify 11 chemokines including IFNγ and IL18.

### Heat-killing tachyzoites, soluble tachyzoite antigen isolation, assay, and treatment

To determine the amount of serum IFNγ that could be induced by live vs heat-killed parasites, freshly isolated tachyzoites of Tg:S1T:Luc:DsRed or NcNc1:Luc:DsRed were left at room temperature or heated to 65 degrees for 60 min at a concentration of 5 × 10^6^ parasites per mL in a total volume of 1 mL. A portion of the heat-killed parasite preparation was then passed through a 0.2 µm filter. Mice (*N* = 4–5 per treatment) were injected with 200 µL (equivalent to 1 × 10^6^ parasites per mouse) of each preparation (live parasites, heat-killed, or heat-killed and filtered) and serum as assayed for IFNγ content 4 h post injection (hpi) as described above. Soluble tachyzoite antigen (STAg) was prepared from multiple strains as described previously (strains used were Tg:S1T:Luc:DsRed, NcNc1:Luc:DsRed, TgME49, and CEPΔHXGPRT:Luc). Soluble and insoluble tachyzoite preparations of 10^9^, 10^8^, 5 × 10^7^, 5 × 10^6^, or 5 × 10^5^ parasites/mL were prepared as follows: monolayers of well-infected confluent human foreskin fibroblast (HFF) cells are washed, scraped, and lysed using a 25 g and 27 g needle and then filtered through a 5 µm filter. Parasites were then counted and adjusted to the desired concentration. Parasite preparations were frozen at −80°C overnight. Sonication was applied to the preparations for 20-second bursts on ice (four times) with 1-min rests (amplitude = 20). After sonication, the samples were centrifuged for 10 min at 13,000 × *g*. The supernatant was removed and kept for injection (STAg), and the insoluble pellet was resuspended in PBS by vortexing. For enzymatic treatments, STAg preparations were incubated with 5 µg/mL of RNase A (Qiagen) and DNase I (Qiagen), or PCR grade proteinase K at 1 mg/mL (Thermo Scientific) for 60 min at 55 degrees, then inactivated at 65 degrees for 10 min. Controls were treated with the same buffer conditions (PBS) and incubated for the same amount of time at the same temperatures. For most experiments, two mice were injected with 1 × 10^6^ parasite equivalents of each preparation, and serum IFNγ was assayed at multiple time points post-injection depending on the experiment (ranging from 4 to 120 h).

### Statistical analysis

All statistical analysis was performed using Prism 7 or 9 (GraphPad Software, Inc). Statistical tests were chosen based on experimental design. Results from flow cytometry experiments were analyzed using one-way analysis of variance (ANOVA) with Tukey’s multiple comparison test unless indicated otherwise in the figure legend. Bioluminescence data were log10-transformed and analyzed using a two-way repeated measure ANOVA (alpha = 0.05) with Sidak’s multiple comparisons test. Cytokine data were analyzed using a Welch ANOVA followed by multiple comparisons which are indicated in the figure legends.

## RESULTS

### Macrophage and dendritic cell populations are not increased at the site of infection or in the spleen 4 h after *T. gondii* and *N. caninum* infection

Our previous work demonstrated that *N. caninum* induces substantially more IL12 and IFNγ as early as 4 hpi compared to *T. gondii* infections (13). Since macrophages and dendritic cells are an important source of IL12 during acute *T. gondii* infections, we hypothesized that the increased IL12 we observed during *N. caninum* infections compared with *T. gondii* infections could be indicative of a differential increase in DCs or macrophages at the site of infection. To determine if *N. caninum* infections resulted in significantly more macrophages or DCs at the site of infection when compared to *T. gondii*, we selected the *N. caninum* strain Nc-1 and two strains of *T. gondii* with known virulence differences in mice. Tg:S1T is a low virulence F1 progeny clone from a type II x III cross that carries “avirulent alleles” for five key virulence loci that impact survival in mice (19). Additionally, we chose the type II Tg:Me49 strain since type-II parasites exhibit higher virulence in mice and frequently cause disease in humans (20). Importantly, type II tachyzoites exhibit stronger associations with CD11c^+^ DC cells than type I, and both type II and III *T. gondii* tachyzoites induce higher migratory frequencies in dendritic cells than type I parasites (21). Since numerous studies have identified CD11c^+^ DCs as a crucial source of IL12 during acute *T. gondii* infections (8, 22, 23), we compared populations of CD11c^+^ DCs (CD45.2+, CD11b-, and MHC II+) between *T. gondii-* and *N. caninum*-infected mice using flow cytometry. Since macrophages are also an important source of IL12 during *T. gondii* infections (24) and depletion of macrophages leads to neosporosis in mice (25), we also compared macrophage populations. To identify macrophages, we employed a similar staining and gating strategy previously described (26) including Ly6G/Ly6C markers (Fig. S1).

**Fig 1:**
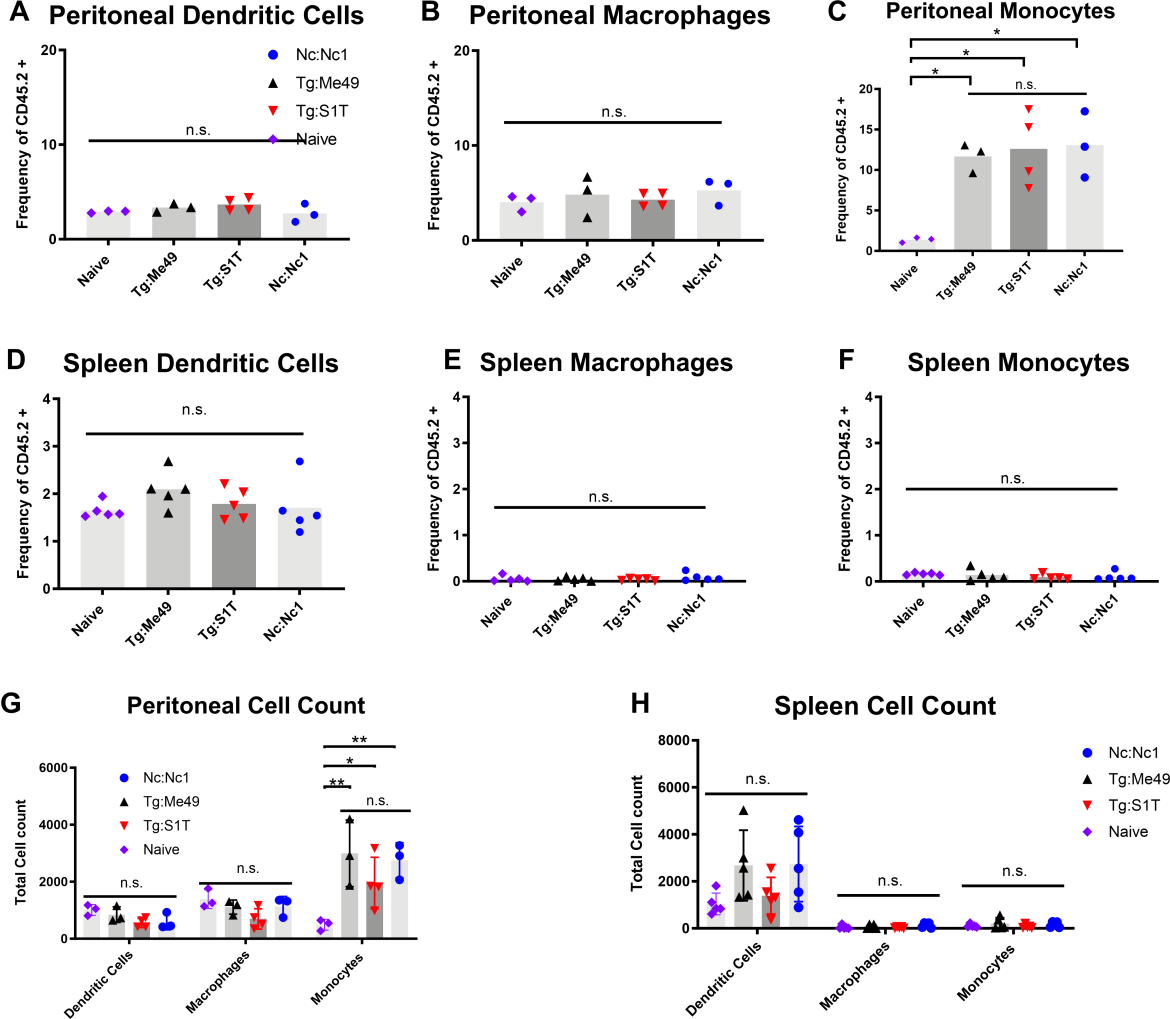
Comparison of peritoneal DC, macrophage, and monocyte populations: 6–8-week-old Balb/C mice were infected with *T. gondii* (Tg:Me49 black, Tg:S1T,red) or *N. caninum* (Nc:Nc1, blue) and compared with uninfected (naïve, purple) samples. Peritoneal and spleen cells were collected and stained with relevant antibody cocktails. Flow cytometric analysis was used to compare cell populations. (**A–F**) *n* = 3 naïve, *n* = 3 Tg:Me49, *n* = 4 Tg:S1T, and *n* = 3 Nc:Nc1. (**G–I**) *n* = 5 naïve, *n* = 5 Tg:S1T, and *n* = 5 Nc:Nc1. Cell populations are frequencies of live leukocytes (frequency of CD45.2+). (A) Peritoneal DCs. (**D**) Spleen DCs. DCs were identified using the gating strategy outlined in Fig. S1 and are defined as CD45+, CD11c+, CD11b−, and MHCII^High^. (**B**) Peritoneal macrophages. (**E**) Spleen macrophages. Macrophages were identified using the gating strategy described in Fig. S1 and are defined as CD45+, CD11b+, Ly6G^Low^, CD19−, and Ly6C^Low^ MHCII^High^. (**C**) Peritoneal monocytes. (F) Spleen monocytes. Monocytes were defined by CD45+, CD11b+, Ly6G^Low^, CD19−, and Ly6C^High^ MHC II^Negative^. (**G**) Absolute cell count of peritoneal cells. (**H**) Absolute cell count of spleen cells. Data were analyzed by one-way ANOVA with Tukey’s multiple comparison test. *P < 0.05, **P < 0.01, and ***P < 0.001.

Peritoneal cells and spleens were collected from infected and uninfected mice for flow cytometry 4 hpi. In the first experiment, we quantified monocytes and macrophages in Naïve, Tg:S1T, and Nc:NC1-infected mice (*N* = 5 mice per condition). To normalize data and compare across multiple experiments, we calculated the cell populations as a frequency of CD45.2, a pan-leukocyte marker. We detected significant decreases in peritoneal macrophages in all infected compared to naïve mice but no differences in macrophage numbers between *T. gondii-* and *N. caninum*-infected mice (Fig. S1B). Peritoneal monocytes were also not different between *T. gondii-* and *N. caninum*-infected mice, although *T. gondii*-infected mice had significantly higher numbers of peritoneal monocytes in this experiment (Fig. S1B). The frequency of monocytes between *N. caninum*-infected and uninfected mice was not statistically significantly different, although they were quantitatively higher in this experiment (Fig. S1B). In spleen cells, monocytes and macrophages were not found to be statistically different in number after *T. gondii* or *N. caninum* infection, although *N. caninum*-infected mice did have significantly fewer monocytes compared to naïve mice (Fig. S1B). Overall this experiment showed no species-specific differences in the recruitment of macrophages or monocytes to the site of infection or the spleen. We repeated this experiment and also quantified dendritic cells along with monocytes and macrophages. In this second experiment, we observed no statistically significant differences in the frequencies (Fig. 1A, B, D, and F) or total number (Fig. 1G and H) of DCs or macrophages across all conditions and sites. However, we did observe a significant (*P* < 0.05) increase in the number of peritoneal monocytes in infected compared to uninfected mice; however, as in the first experiment in Fig. S1B, there were no significant differences in monocyte frequency between *T. gondii* and *N. caninum* infections (Fig. 1C and G). As with macrophages and DCs, there were no significant differences across all spleen samples (Fig. 1F and H), although the frequency of monocytes and macrophages was lower in this experiment overall compared to the first experiment in Fig. S1B. These data suggest that monocyte populations are not significantly increased in *N. caninum* infections compared to *T. gondii,* but both infections significantly recruit monocytes to the infection site 4 h post-infection. While there was some inter-experiment variability in cell-type frequency in both of these trials, the important finding is that neither experiment showed any evidence of differential cell recruitment to the peritoneum nor spleen based on the infecting parasite species. Together these data indicate that *T. gondii* (in both experiments) and *N. caninum* (in 1 of 2 experiments) infections induce similar populations of monocytes in the peritoneal cavity, but populations of DC and macrophages do not increase above naïve levels in the same timeframe despite substantially different inflammatory cytokine production profiles (13).

### Mice lacking T cells or NKT cells still produce immediate interferon gamma in response to *N. caninum* and exhibit either partial or complete early control of *N. caninum* compared to *T. gondii*

**Fig 2:**
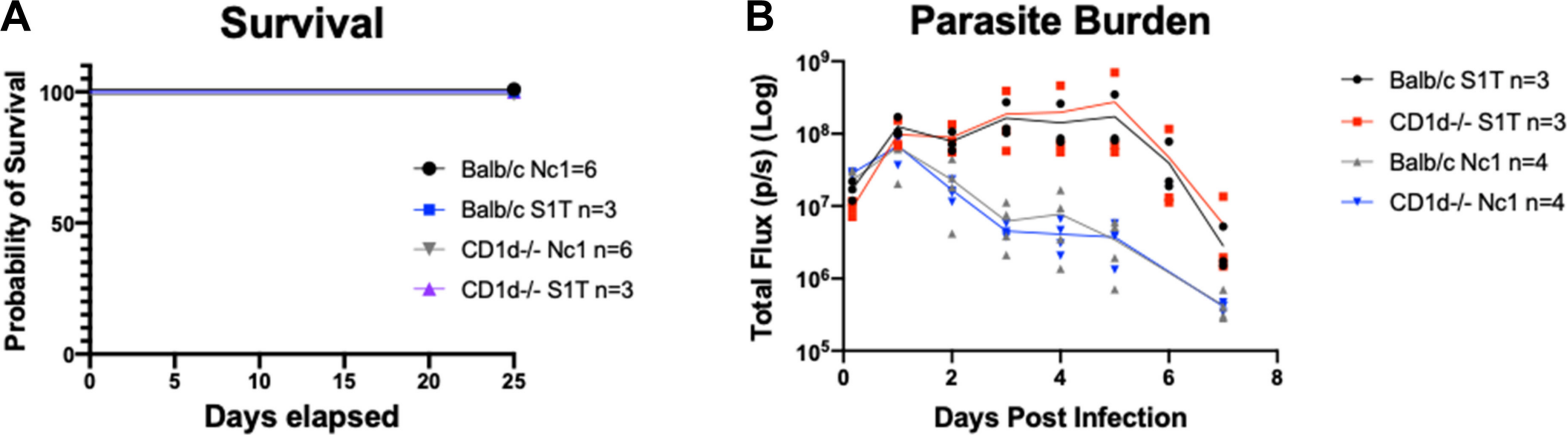
Comparison of *T. gondii* and *N. caninum* infections in Cd1d^−/−^ mice. Cd1d^−/−^ mice or Balb/C mice were infected with 10^6^ tachyzoites of either *N. caninum* Nc1:Luc or *T. gondii* S1T:Luc. (**A**) Survival graph comparing Balb/C infected with *N. caninum* NC1:Luc (black circle *n* = 6), or *T. gondii* S1T:Luc (blue square *n* = 3) and Cd1d^−/−^ mice infected with *N. caninum* NC1:Luc (gray triangle *n* = 6) or *T. gondii* S1T:Luc (purple triangle, *n* = 3). (**B**) Parasite burden measured with bioluminescent imaging comparing Balb/C infected with NC1:Luc (gray triangle *n* = 4), or S1T:Luc (black circle *n* = 3) and Cd1d^−/−^ mice infected with NC1:Luc (blue triangle *n* = 4) or *T. gondii* S1T:Luc (red square, *n* = 3). Log10-transformed bioluminescent imaging data were analyzed using a two-way repeated measure ANOVA (alpha = 0.05) with Sidak’s multiple comparisons test, *P < 0.05, **P < 0.01, and ***P < 0.001. (**C**) Serum IFNγ production 4 h post-infection with the indicated parasite species (Tg or Nc) and mouse genetic background. Data were analyzed using a two-way repeated measure ANOVA (alpha = 0.05) with Sidak’s multiple comparisons test, *P < 0.05, **P < 0.01, and ***P < 0.001. *n* = 6 for all conditions except for TgCD1^−/−^ where *n* = 3.

To begin to narrow down the cell types that might be responsible for immediate production of IFNγ in response to *N. caninum* infection, we used an assorted panel of knockout mice with deficiencies in IFNγ-producing cells, which include NKT cells, NK cells, and T cells. To test the requirement of NKT cells for immediate IFNγ production and control of parasite proliferation in NKT-deficient mice, we used CD1d-deficient mice. CD1 family proteins are structurally similar to MHC class I proteins and also function as antigen-presenting molecules that are important during innate and adaptive immunity. CD1 family proteins are known to present lipid and glycolipid antigens to NKT cells. iNKTs require CD1d for development; therefore, CD1d-deficient mice (CD1d^−/−^) lack iNKTs (27). Balb/C (WT) or Balb/C mice lacking iNKT cells (Cd1d^−/−^) were infected with 10^6^ Nc1:Luc or 10^6^ TgS1T:Luc tachyzoites, and bioluminescent imaging was performed as previously described (13). We did not observe any differences in survival between WT and CD1d^−/−^ mice in either *T. gondii* or *N. caninum* infections (Fig. 2A). Additionally, we did not observe differences in parasite proliferation between WT and CD1d^−/−^ infections (Fig. 2B). To determine if the loss of Cd1d-restricted NKT cells would impact IFNγ production, we tested serum samples from WT and CD1d^−/−^ mice 4 h after infection and analyzed samples for IFNγ via luminex. Again, we saw no significant differences in mice lacking iNKTs cells to produce IFNγ in response to *N. caninum* compared to WT infections (data in Fig. 5A). Although we determined that CD1d-restricted NKT cells are not responsible for the immediate production of IFNγ during *N. caninum* infections, we could not rule out NKT cells with Cd1d-independent restriction.

**Fig 3:**
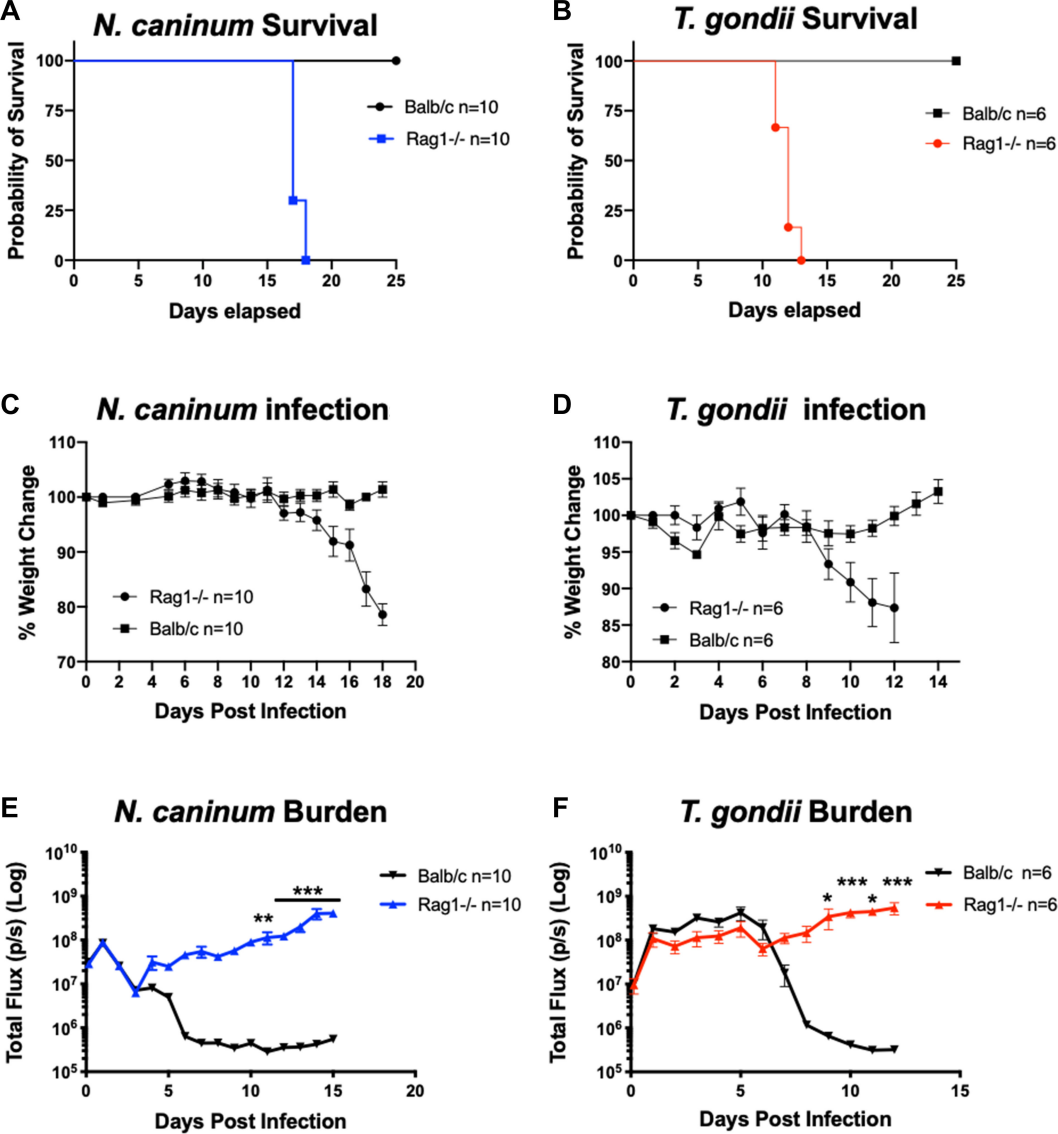
*In vivo* bioluminescence imaging comparing *N. caninum* and *T. gondii* acute infection in Rag1-deficient (Rag^−/−^) or WT Balb/C mice Rag1 knockout mice (Rag1^−/−^) or Balb/C mice were infected with 10^6^ tachyzoites of either *N. caninum* Nc1 or *T. gondii* S1T. (**A**) Survival graph of *N. caninum-*infected Rag^−/−^ (blue, *n* = 10) or Balb/C mice (black *n* = 10). Data shown are combined from three separate experiments. (**B**) Survival graph of *T. gondii-*infected Rag^−/−^ (*n* = 6) or Balb/C mice (*n* = 6). Data shown are from two separate experiments. (**C and D**) Percent weight change of (C) *N. caninum-*infected Rag1^−/−^ (*n* = 10) and Balb/C (*n* = 10) mice or (D) *T. gondii-*infected Rag^−/−^ (*n* = 6) and Balb/C (*n* = 6) mice. (**E**) Parasite burden of Nc1:Luc infection in either Balb/C (black *n* = 10) or Rag^−/−^ (blue, *n* = 10). (**F**) Parasite burden of TgS1T:Luc infections in Balb/C (black *n* = 6) or Rag^−/−^ (red, *n* = 6). Bioluminescent imaging data were analyzed using a two-way repeated measure ANOVA (alpha = 0.05) with Sidak’s multiple comparisons test, *P < 0.05, **P < 0.01, and ***P < 0.001.

We next compared parasite proliferation and cytokine production in Rag-deficient mice (Rag1^−/−^) that lack both T and NKT cells but still exhibit a robust NK cell response (28). Over the entire course of the experiment, Rag-deficient mice were highly susceptibility to both *T. gondii* and *N. caninum* infections, but *T. gondii-*infected mice succumbed earlier in infection (11–13 days post infection (dpi), Fig. 3B and D, *n* = 6) compared to *N. caninum-*infected mice (17–18 dpi, Fig. 3A and C, *n* = 10). Despite this clear increase in susceptibility to both parasite species (WT mice survived infection of both species), there were no changes to host susceptibility early in infection (between days 1 and 8), where we saw no statistically significant difference in parasite burden comparing RAG1^−/−^ to WT mice with either *T. gondii* or *N. caninum* (Fig. 3E and F). Importantly, we observed early control of *N. caninum* in both WT and RAG1^−/−^ mice, as indicated by a decline in *N. caninum*-derived luciferase signal on the second day of infection (Fig. 3E). After the initial control phase (0–3 dpi), *N. caninum* infections rebounded in RAG^−/−^ mice and parasites exhibited increased proliferation until mice succumbed to infection. In contrast, *T. gondii* infections lacked the initial control phase in both Rag^−/−^ and WT mice, and control of proliferation was observed 6–7 dpi in WT and not Rag^−/−^ mice (Fig. 3F). Rag-deficient mice were equally capable of producing robust IFNγ 4 h after *N. caninum* infections as WT mice (Fig. 5A; IFNγ data shown for multiple knockout mouse experiments). Together these results demonstrate that T cells are not required for control of *N. caninum* proliferation during the early phase of infection.

### NK cell-deficient Rag2^−/−^ γC^−/−^ knockout mice fail to produce IFNγ or control *N. caninum* and *T. gondii* infections

**Fig 4:**
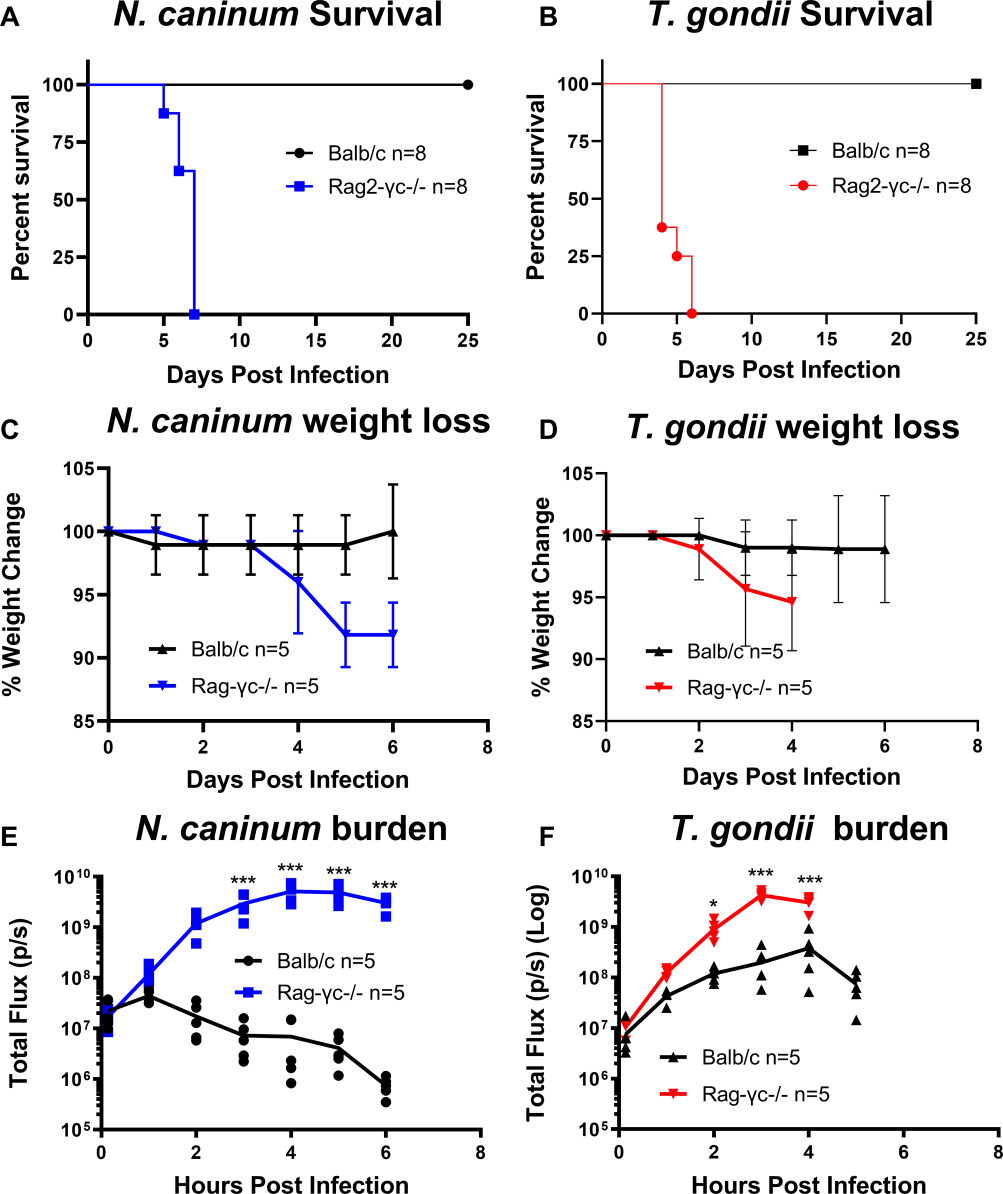
*In vivo* bioluminescence imaging comparing *N. caninum* and *T. gondii* acute infection in Rag2^−/−^ γC^−/−^ or Balb/C mice. Rag2^−/−^ γC^−/−^ (Rag-γc^−/−^) mice or Balb/C mice were infected with 10^6^ tachyzoites of either *N. caninum* Nc1:Luc or *T. gondii* S1T:Luc. (**A**) Survival graph of *N. caninum-*infected Rag-γc^−/−^ (blue, *n* = 8) or Balb/C mice (black *n* = 8). The data shown are combined from two separate experiments. (**B**) Survival graph of *T. gondii-*infected Rag-γc^−/−^ (red, *n* = 8) or Balb/C mice (black, *n* = 8). The data shown are from two separate experiments. (**C and D**) Percent weight change of (C) *N. caninum-*infected Rag-γc^−/−^ (*n* = 5) and Balb/C (*n* = 5) mice data shown is representative of two independent experiments. (**D**) Percent weight change of *T. gondii-*infected Rag2-γc^−/−^ (*n* = 5) and Balb/C (*n* = 5) mice. The data shown are representative of two independent experiments. (E) Parasite burden of Nc1:Luc infection in either Balb/C (black *n* = 5) or Rag2-γc^−/−^ (blue, *n* = 5). (**F**) Parasite burden of TgS1T:Luc infections in Balb/C (black *n* = 5) or Rag2-γc^−/−^ (red, *n* = 5). The data shown are representative of two independent experiments. Bioluminescent imaging data were analyzed using a two-way repeated measure ANOVA (alpha = 0.05) with Sidak’s multiple comparisons test, *P < 0.05, **P < 0.01, and ***P < 0.001.

**Fig 5:**
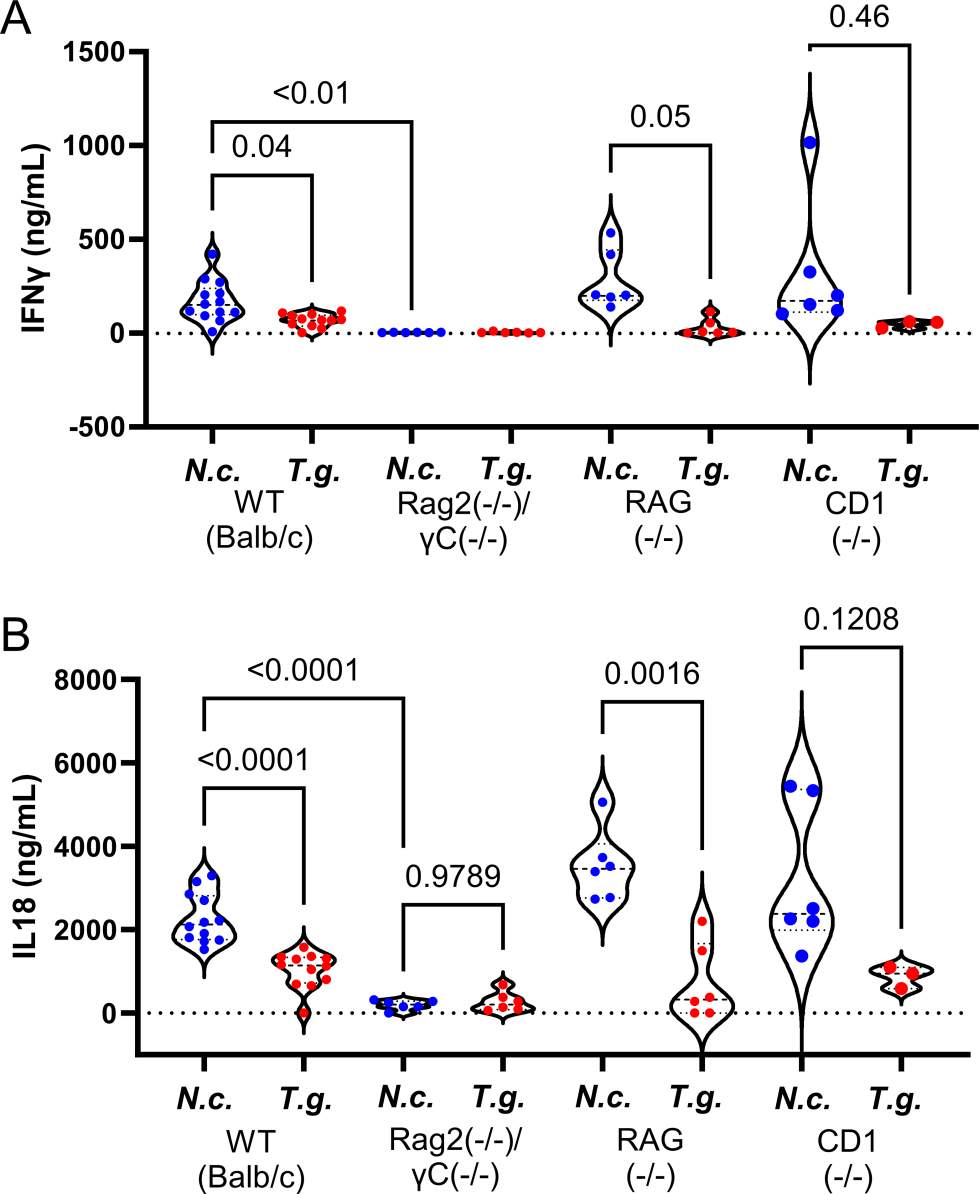
Serum IFNγ (**A**) and IL18 (**B**) concentration 4 h after infection with either *T. gondii* or *N. caninum*. Six-week-old Balb/C, Rag^−/−^, Rag2^−/−^/γc^−/−^, or Cd1d^−/−^ mice were injected IP with 10^6^ tachyzoites of either (A) Nc1:Luc or (B) TgS1T:Luc tachyzoites. Blood samples were collected 4 h after infection and were analyzed using a mouse cytokine/chemokine multiplex assay (Luminex). Statistical analysis was determined using a one-way Brown-Forsythe ANOVA to account for differences in SD across groups followed by Dunnett’s T3 multiple comparisons. Comparisons to be used were planned in advance and were *N. caninum*-infected WT mice compared all knockouts, as well as *N. caninum* vs *T. gondii* for each mouse strain (WT and all knockouts). *P* values are indicated above each comparison.

To determine the role of NK cells in the early control of *N. caninum* infections, we compared infections of *N. caninum* and *T. gondii* in mice lacking both a functional Rag2 gene and the γ chain of the interleukin 2 receptor (Rag2^−/−^ γC^−/−^) mice. Rag2^−/−^ γC^−/−^ mice lack functional T and B cells like Rag1^−/−^ mice but additionally lack functional NK cells. Based on our findings that Rag1^−/−^ mice lacking all T cells, including NKT cells, were still able to control *N. caninum* infections (Fig. 3) and produce immediate IFNγ (Fig. 5A), we hypothesized that infections in Rag2^−/−^ γC^−/−^ mice lacking NK cells would result in uncontrolled proliferation and the loss if IFNγ production. Mice were infected as described above, and bioluminescent imaging was performed throughout the infections, and serum IFNγ was analyzed 4 hpi by Luminex analysis (Data Set S1). Rag2^−/−^ γ^−/−^ mice succumbed to both *T. gondii* infection and *N. caninum* within 6 days after infection (Fig. 4A and B). Both *T. gondii* and *N. caninum* proliferated significantly more in the Rag2^−/−^ γC^−/−^ mice compared to the wild type resulting in acute host morbidity (Fig. 4C through F). We observed no IFNγ production in either *N. caninum* or *T. gondii* infections in Rag2^−/−^ γC^−/−^ mice (Fig. 5A), and when these data are contrasted with data from RAG1^−/−^ mice, it strongly supports a role for NK cells as being the critical source of early IFNγ during *N. caninum* infections, and this early response is capable of controlling *N. caninum* proliferation as early as 4 h post-infection. While NK cells are also critical for the control of *T. gondii* and mouse survival, they do not appear to be activated as quickly after infection with *T. gondii* compared to *N. caninum* (13). In addition to IFNγ, we also quantified and statistically analyzed IL18 levels in wild-type and knockout mice (Fig. 5B) given the potential role played by this cytokine in the rapid production of IFNγ (29). We found that serum IL18 levels tracked consistently with IFNγ levels, being more highly induced in *N. caninum* infections compared to *T. gondii* (Fig. 5B) in WT, RAG(−/−), and CD1(−/−) mice. However, there was still detectable IL18 in Rag2^−/−^ γC^−/−^ mice (Fig. 5B), while IFNγ was undetectable in these mice (Fig. 5A). This suggests that either IL18 is not responsible for driving immediate IFNγ production in response to *N. caninum*, or it is below a threshold for induction of IFNγ production in Rag2^−/−^ γC^−/−^ mice. Data from nine other chemokines/cytokines from the 11-plex Luminex assay at 4 h post-infection can be found in Data Set S1.

### *N. caninum* infections exhibit significantly more IFNγ+ peritoneal cells 4 h after infection compared to *T. gondii-*infected and naïve mice

**Fig 6:**
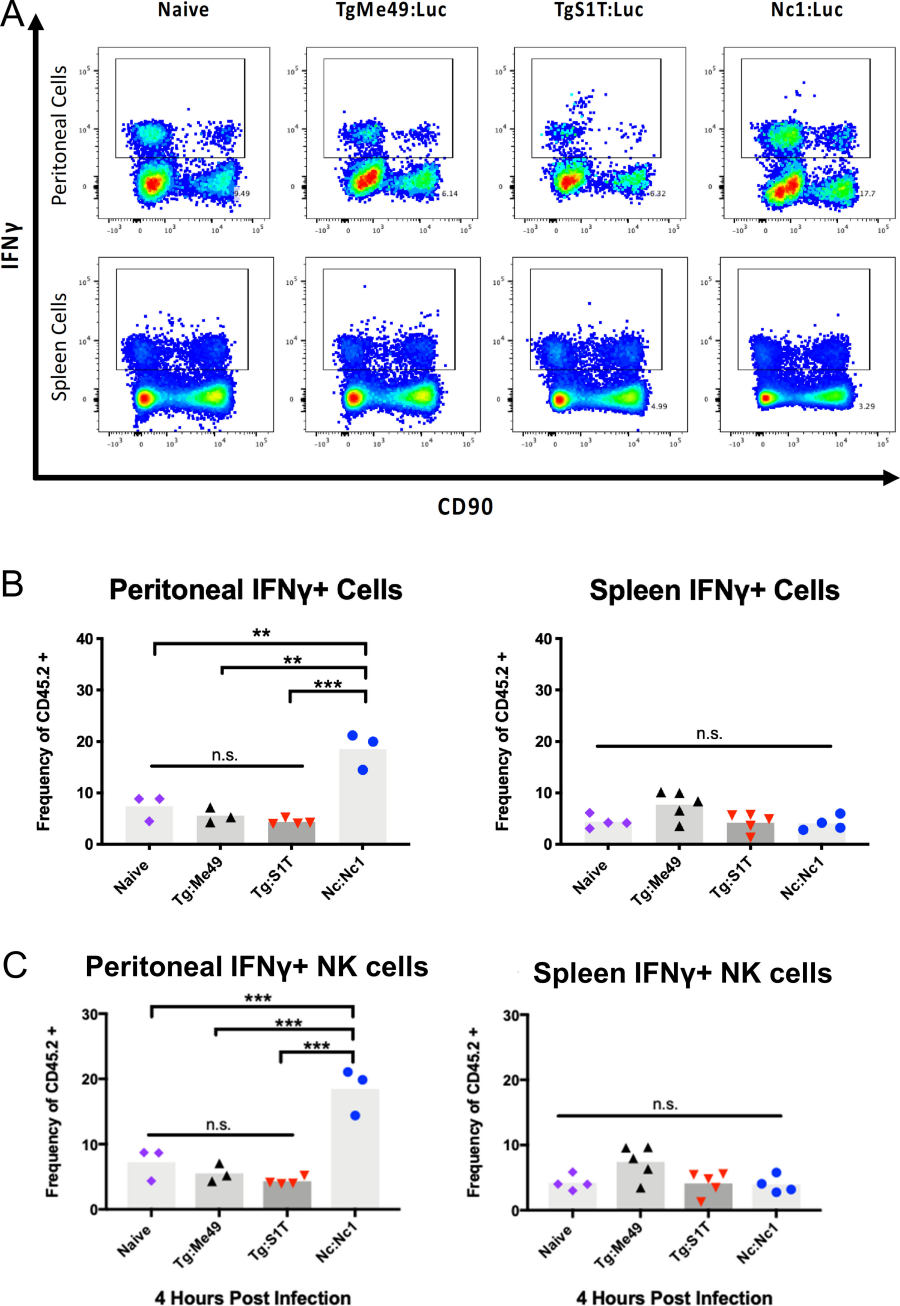
Determination of the cellular source of IFNγ after infection with *T. gondii* and *N. caninum*. Mice were infected with *T. gondii* (Tg:Me49, black, Tg:S1T, red) or *N. caninum* (Nc:Nc1, blue) and compared with uninfected (naïve, purple) samples. Peritoneal and spleen cells were collected and stained with relevant antibody cocktails, and intracellular cytokine staining was performed. (**A**) Representative flow plots of IFNγ+ cells were identified using the gating strategy outlined in Fig. S2 to quantify IFNγ+/CD45+ cells. (**B**) Frequency of peritoneal CD45+ live cells that are IFNγ+ in PECs (left) and spleen (right). *N* = 3 mice per condition. (**C**) In a separate experiment, the frequency of IFNγ+ NK cells was determined in mice infected with the same *T. gondii* strains using the gating strategy outlined in Fig. S2. NK cells were identified as the frequency of live CD45+ cells that were also CD90−/CD3− and DX5+ in PECs and spleen (*N* = 3 mice per condition). Data for both experiments were analyzed using one-way ANOVA with Tukey’s multiple comparison test to identify significance. *P < 0.05, **P < 0.01, and ***P < 0.001.

Our previous work described MyD88-dependent production of IFNγ that was detected both at the site of infection and in the serum during the first few hours of *N. caninum* infection, but the cells producing IFNγ immediately after infection remained unknown (13). Therefore, we sought to compare populations of IFNγ-producing cells with the expectation that the number of peritoneal cells producing IFNγ would be significantly elevated in *N. caninum* infections compared to *T. gondii* and naïve mice. We infected Balb/C mice via IP injection with 10^6^ *N. caninum* (Nc:Nc1) or *T. gondii* (Tg:S1T or Tg:Me49) tachyzoites. To quantify the number of IFNγ-producing cells, we performed intracellular cytokine staining and flow cytometry on *T. gondii-* and *N. caninum-*infected or naïve mouse samples (gating as in Fig S2A; Fig. 6A). To compare across multiple experiments, we calculated the IFNγ+ cells as the percent of live leukocytes (CD45.2+; Fig. 6A). We found that the frequencies of IFNγ+ CD42+ cells were significantly elevated only in peritoneal cells from *N. caninum*-infected mice at 4 h post-infection (Fig. 6B). In contrast, there were no differences in the frequencies of IFNγ+ peritoneal cells between naïve and *T. gondii*-infected mice (Fig. 6B).

### The majority of IFNγ+ peritoneal and spleen cells after *T. gondii* or *N. caninum* infection are DX5+ natural killer cells

As a first step toward identifying the cells in both the peritoneum and spleen that produce IFNγ in response to *N. caninum*, we infected Balb/C mice with 10^6^ tachyzoites of *N. caninum* NC1 strain and used flow cytometry and intracellular cytokine staining on cells harvested at 10 h post-infection. As a control, we also infected mice with 10^6^ *T. gondii* S1T tachyzoites and harvested cells at the same time point. Given that NK cells are known to produce IFNγ in response to *T. gondii* infection (1), we focused our flow cytometry panel on this cell type. The heterogeneity of NK marker expression has made NK cells more elusive to characterize than other innate cells (30), and flow cytometry panels utilize a number of NK lineage-exclusionary markers like CD3 and TCRβ combined with species/strain-appropriate NK markers to evaluate NK cell populations (31). To identify NK cells in the present study, we identified IFNγ-producing NK cells as CD45.2+, IFNγ+, DX5+, TCRβ−, CD90−, and CD3− (Fig. S2A). We found that in all *N. caninum-* and *T. gondii*-infected mice, the vast majority of IFNγ+ cells were DX5+ NK cells in both peritoneal exudate cell and spleen (Fig. S2B), and the relative frequencies of these cells did not differ depending on the species used to infect the animal (Fig. S2B). Overall these data show that early IFNγ production in mice in response to *N. caninum* is driven primarily by NK cells, similar to *T. gondii*.

### *N. caninum*-infected mice have higher numbers of IFNγ+ NK cells at the site of infection compared to naïve and *T. gondii*-infected mice at 4 h post-infection

Given the known differences in the timing of IFNγ production in response to *N. caninum* infection (13) as well as the significantly higher frequency of peritoneal IFNγ+ cells 4 h post-infection in *N. caninum*-infected mice compared to those infected with *T. gondii* (Fig. 6B), we used a follow-up experiment to quantify the numbers of IFNγ+ NK cells in peritoneal and spleen cells from mice infected with each species. Similar to the experiments described above, we compared peritoneal and spleen cells from Balb/C mice-infected IP with 10^6^ *N. caninum* (Nc:Nc1) *T. gondii* (Tg:S1T) or Tg:Me49) with naïve mice. Our analysis revealed that peritoneal NK cells (CD45.2+/IFNγ+/DX5+, TCRβ−/CD90−/CD3−) in *N. caninum* infections were significantly elevated compared to *T. gondii* and naïve samples (Fig. 6C). As with other cell populations, we saw no significant difference in the frequency of spleen NK cells (Fig. 6C). Taken with data shown in Fig. S2, these data show that a likely reason for immediate control of *N. caninum* in mouse infections is the induction of IFNγ production by NK cells at the site of infection. While this has a similar role for NK cells compared to that in *T. gondii* infection, the critical difference is that this response is launched faster in *N. caninum* compared to *T. gondii*, a likely contributor to the highly effective control of *N. caninum* in the mouse.

### Immediate IFNγ production by *N. caninum* requires a parasite-derived protein component that is heat labile

**Fig 7:**
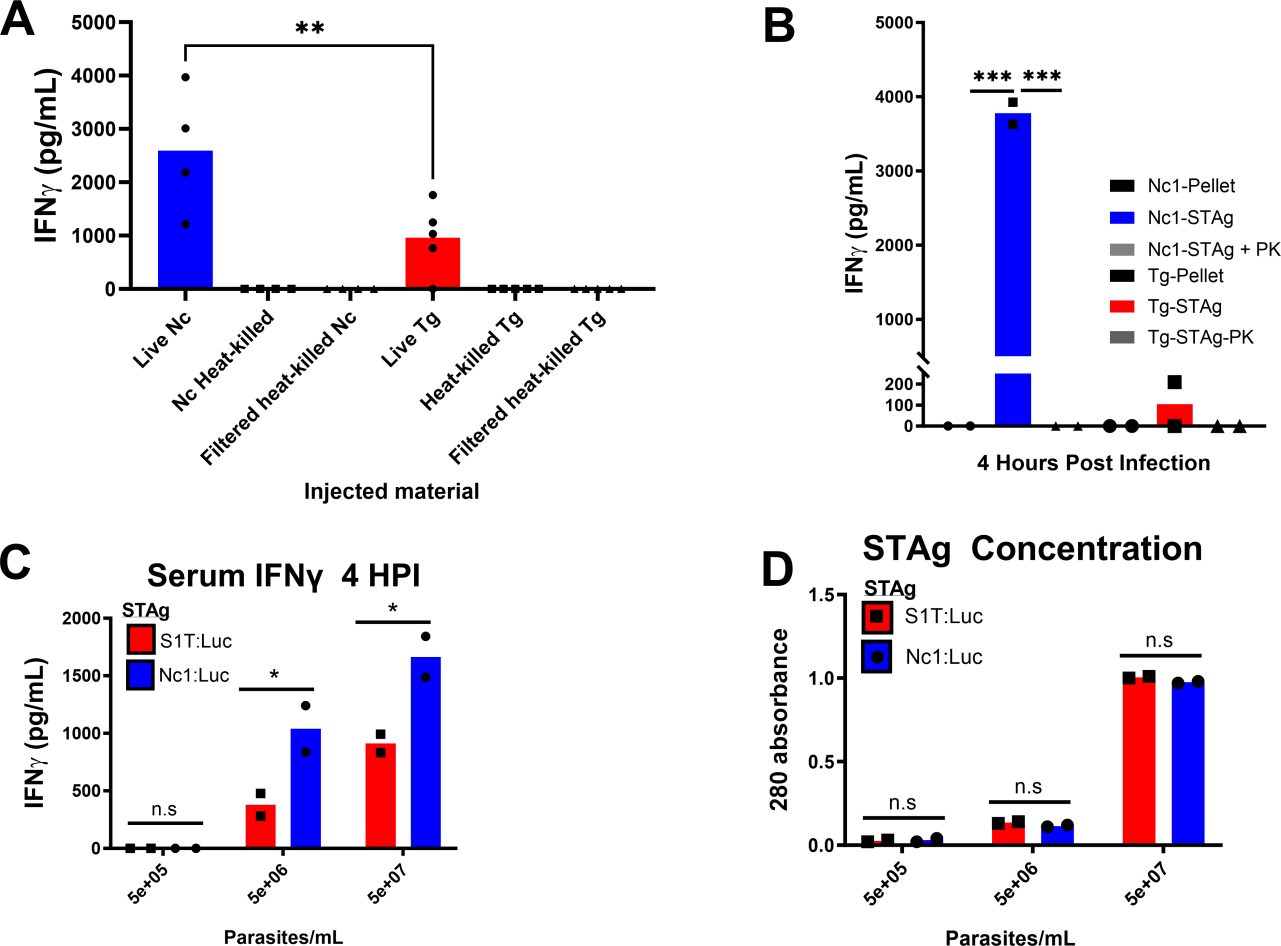
*N. caninum* STAg induces more serum IFNγ compared to equivalent amounts of *T. gondii* STAg. (A) Heat-killed *N. caninum* and *T. gondi* were injected into mice, and serum IFNγ was assayed 4 h post-injection. Only live parasites induced detectable IFNγ, and heat-killed *N. caninum* induced significantly more serum IFNγ compared to *T. gondii* (**: *P* < 0.01). (B) *N. caninum* and *T. gondii* STAg were isolated from the same number of freshly lysed tachyozites and tested for their ability to induce serum IFNγ in mice. Only STAg, and not the insoluble pellets, from either preparation induced detectable IFNγ 4 h post-injection. Proteinase K treatment of STAg from both species eliminated IFNγ induction and *N. caninum* STAg induced significantly more IFNγ compared to *T. gondii* STAg (*P* < 0.0001). (C) *N. caninum* STAg induces significantly (*: *P* < 0.05) more serum IFNγ compared to *T. gondii* in mice after i.p. injection at multiple parasite-equivalent doses. Both *N. caninum* and *T. gondii* STAg induction of IFNγ is dose dependent. (D) Estimated protein concentration based on absorbance at 280 nm of *N. caninum* and *T. gondii* STAg preparations injected into mice in panel C, showing similar amounts of protein for preparations from each species.

To begin investigating the parasite factor(s) responsible for immediate IFNγ induction, we compared *T. gondii* and *N. caninum* tachyzoite preparations for *in vivo* IFNγ production. We compared IFNγ levels 4 h after injection of 10^6^ live, heat-inactivated (heat-killed), or heat-killed and filtered tachyzoites in a head-to-head comparison between *T. gondii* and *N. caninum*. Serum was collected 4 h after injection, and IFNγ concentrations were measured by enzyme-linked immunosorbent assay (ELISA). As we saw in other infections with live 10^6^ tachyzoites, we observed significantly higher (*P* < 0.05) IFNγ levels in *N. caninum* compared to *T. gondii* (Fig. 7A). Neither *T. gondii* or *N. caninum* heat-killed or filtered heat-killed preparations induced IFNγ 4 h after injection (Fig. 6A). These results demonstrate that the IFNγ-inducing parasite molecule(s) are heat labile. Next, we injected mice with either STAg or insoluble tachyzoite (pellet) preparations and measured IFNγ production. We found that NcSTAg induces IFNγ within 4 h post-injection while the pellet fractions do not (Fig. 7B; Fig. S3A). As expected, TgSTAg induced detectable IFNγ at 4 h, albeit ~400-fold less than that induced by *N. caninum* STAg in this particular experiment (Fig. 7B). This species-specific difference was still apparent in other experiments (see below) but was less pronounced. Treatment of either NcSTAg or TgSTAg with proteinase-K abolished IFNγ production *in vivo* (Fig. 7B; Fig. S2A), while treatment with an RNase/DNase cocktail did not abolish the IFNγ-inducing activity of NcSTAg (we did not test RNase/DNase on TgSTAg; Fig. S3B). Together, these results suggest that a soluble protein component in *N. caninum* tachyzoite lysate is activating immediate IFNγ production in mouse infections, and they do so with greater potency than similar preparations from *T. gondii* in a manner consistent with what is observed with live parasites.

Previous work has demonstrated that *N. caninum* is only able to overcome host protective immunity at extremely high doses, often in excess of 2 × 10^7^ (32). *T. gondii* pathogenicity has been well described for clonal types that are predominant in Western Europe and the United States. Clonal types I, II, and III have lethal doses (LD) of LD100 of 1, LD50 10^3^ and 10^5^, respectively (33). In order to address how different dosages of STAg might impact the immediate induction of IFNγ, we analyzed serum from mice injected with STAg prepared from 5 × 10^5^, 5 × 10^6^, or 5 × 10^7^ tachyzoites of either *T. gondii* (TgS1T:Luc) or *N. caninum* (Nc1:Luc). Sample protein concentrations were estimated by measuring 280 nm OD (Fig. 7D) as previously described (34). Balb/C mice (8 weeks old) were injected with 10^5^, 10^6^, or 10^7^ parasite equivalent dosages of STAg (200 µL). At 10^6^ and 10^7^ tachyzoite equivalent STAg injections, we observed *N. caninum* STAg induced significantly more than the equivalent dosages of *T. gondii* STAg (Fig. 7C). We did not detect IFNγ production in mice injected with 10^5^ parasite equivalents of TgSTAg or NcSTAg (Fig. 7C). In comparisons with more virulent *T. gondii* strains at doses that are normally lethal (5 × 10^6^ p/mL), we observed both Me49 (type II) and CTG (Type III) TgSTAg preparations induced significantly less (*P* < 0.001) than equivalent concentrations of NcSTAg (Fig. S3C). Taken together, these data indicate that STAg-induced IFNγ production is dose dependent and increases at higher concentrations of STAg, and *N. caninum* STAg induces significantly more IFNγ when compared with STAg from multiple strains of *T. gondii*.

## DISCUSSION

Group 1 Innate lymphoid cells include NK cells and ILC1s, and both have been identified as important innate sources of IFNγ during *T. gondii* infections (17, 35). Recent advances in NK cell biology have revealed significant diversity in NK cell phenotype and function using transcription factors, NK marker expression, tissue specificity, and effector function (35). Substantial efforts have been made to determine NK subtypes in numerous tissues, including the liver, skin, the uterus, submandibular glands, the spleen, kidneys, and circulation, yet few have examined peritoneal cells at steady state or early after infection (17, 36–38). Here, using two parasite species with distinct host compatibilities, we demonstrate a role for NK cells in producing early and effective IFNγ in response to *N. caninum* infection. Functional assays would be necessary to determine which NK subtype(s) is responsible because NK cell designations are often based on functionality (38). The fact that NK cells are required for IFNγ production in response to *N. caninum* is consistent with the critical role played by this cell type in the production of IL12-dependent IFNγ in *T. gondii* infection. As shown in our prior work (13) and in the current manuscript (Fig. 5), serum IFNγ can be detected in *N. caninum*-infected mice at earlier time points than *T. gondii*-infected mice, and this effect is MyD88 and IL12 dependent. *T. gondii* is either capable of suppressing these early responses or avoiding detection during the first 24–48 of infection. While the mechanisms for this are still unclear, it could occur by either poor recognition of *T. gondii* ligands by pattern recognition receptors expressed on macrophages or dendritic cells such as TLR11 (13, 22) or active suppression of DC or macrophage IL12 production during the early phase of infection. Our data also show that IL18 levels are also significantly higher in *N. caninum*-infected mice at 4 h post-infection, and IL18 levels generally track with IFNγ (Fig. 5; Data Set S1). IL18 is known to enhance IL12-dependent IFNγ production in mice (39), and these data suggest that early induction of IL18 (along with IL12 as we have shown previously [13]) may help potentiate the *N. caninum*-driven IFNγ response.

NKT cells are also important sources of IFNγ during apicomplexan infections, and previous work investigates NKT cell cytokine production beginning at 7 days after *T. gondii* infections (40, 41). However, NKT cells appear to not be important in early responses to *N. caninum*. We previously determined that immediate production of IFNγ is MyD88 dependent, and an important future direction of this work is to identify the TLR mechanism that results in NK cell IFNγ production. This may be via direct stimulation or dependent on an accessory cell that is required for NK activation. This, combined with our previous findings that IFNγ^−/−^ mice are more susceptible to *N. caninum* proliferation, suggests that *N. caninum* infections induce specific, cell-mediated immune responses driven by IFNγ, and in the absence of IFNγ-driven cell mediate mechanisms, *N. caninum* proliferation is unmitigated.

The major component of *T. gondii* STAg responsible for the induction of IL12 and subsequently IFNγ *in vivo* is the TLR11 ligand profilin (22). However, our prior work (13) determined that the early induction of IFNγ by *N. caninum* is not exclusively due to *T. gondii* profilin since parasites expressing overexpressed versions of these proteins did not induce more IFNγ than their wild-type counterparts (13). NcSTAg was significantly more potent than TgSTAg at inducing IFNγ in mice across multiple preparations, suggesting either that *T. gondii* STAg has an IFNγ suppressive effect or NcSTAg contains a factor(s) not present at all in TgSTAg or of significantly different abundance. This is the first demonstration that the difference in the innate response to *N. caninum* and *T. gondii* is driven, at least in part, by differences in responses to parasite antigen rather than infection by live parasites. Very preliminary efforts to fractionate NcSTAg from moderate numbers of parasites and test the ability of fractions to induce IFNγ in mice have been carried out with limited success, and therefore, scaling these preparations up to permit direct purification and identification of IFNγ-inducing proteins in NcSTAg may be a valid approach moving forward. One issue is developing a reliable *in vitro* assay. NcSTAg can induce IFNγ when used to treat primary mouse bone marrow and spleen cells (42) but only at very high doses compared to the minimum concentration of live parasites needed for the same effect (42). It will also be of interest to examine IL18 production in response to TgSTAg and NcSTAg and to determine if this differs between species as well. It is known that *T. gondii* can induce inflammasome activation in CD4+ T cells in the absence of TLR11 (43), and *N. caninum* induces IL18 and IL1β secretion in mouse bone marrow-derived macrophages *in vitro* (44). This is an intriguing model given that IL18 levels are higher in *N. caninum*-infected mice as early as 4 h post-infection (Fig. 5B).

Taken together these data provide further insight into the mechanisms of host-range differences between *N. caninum* and *T. gondii*, specifically in the mouse model. The evolutionary relationship between *T. gondii* and rodent species is profound and critical for the effective transmission and expansion of the parasite in the feline definitive host through the production of environmentally stable oocysts (45, 46). This relationship has resulted in a clear species-specific molecular arms race between *T. gondii* effectors belonging to the ROP2 superfamily and mouse immunity-related GTPase proteins that are capable of mediating IFNγ-induced vacuolar destruction (47–50). But it appears that at least some of the divergence in host range between *N. caninum* and *T. gondii* is due to dramatic differences in the timing and quantity of IFNγ induction, suggesting that another substrate for co-evolution in the *T. gondii*-mouse cycle was either active suppression or evasion of immediate IFNγ responses. Our data indicate that while the IFNγ-producing cells are likely the same for these responses (NK cells), they respond differently to infection and antigen preparations from *N. caninum*.

## SUPPLEMENTAL MATERIAL

Data Set S1 msphere.00255-24-s0001.xlsx Luminex analysis of 11 cytokines and chemokines from mice infected with *T. gondii* or *N. caninum*.

Supplemental material msphere.00255-24-s0002.pdf Figures S1 to S3 and legend for Data Set S1.

ASM does not own the copyrights to Supplemental Material that may be linked to, or accessed through, an article. The authors have granted ASM a non-exclusive, world-wide license to publish the Supplemental Material files. Please contact the corresponding author directly for reuse.
