# Supplementary material for: Mouse innate resistance to Neospora caninum infection is driven by early production of IFNγ by NK cells in response to parasite ligands
Source: mSphere. 2024 Oct 24;9(11):e00255-24. doi: 10.1128/msphere.00255-24 (PMC11580461; doi:10.1128/msphere.00255-24)
Supplement: Supplemental material — Figures S1 to S3 and legend for Data Set S1. [file msphere.00255-24-s0002.pdf]

Figure S1:

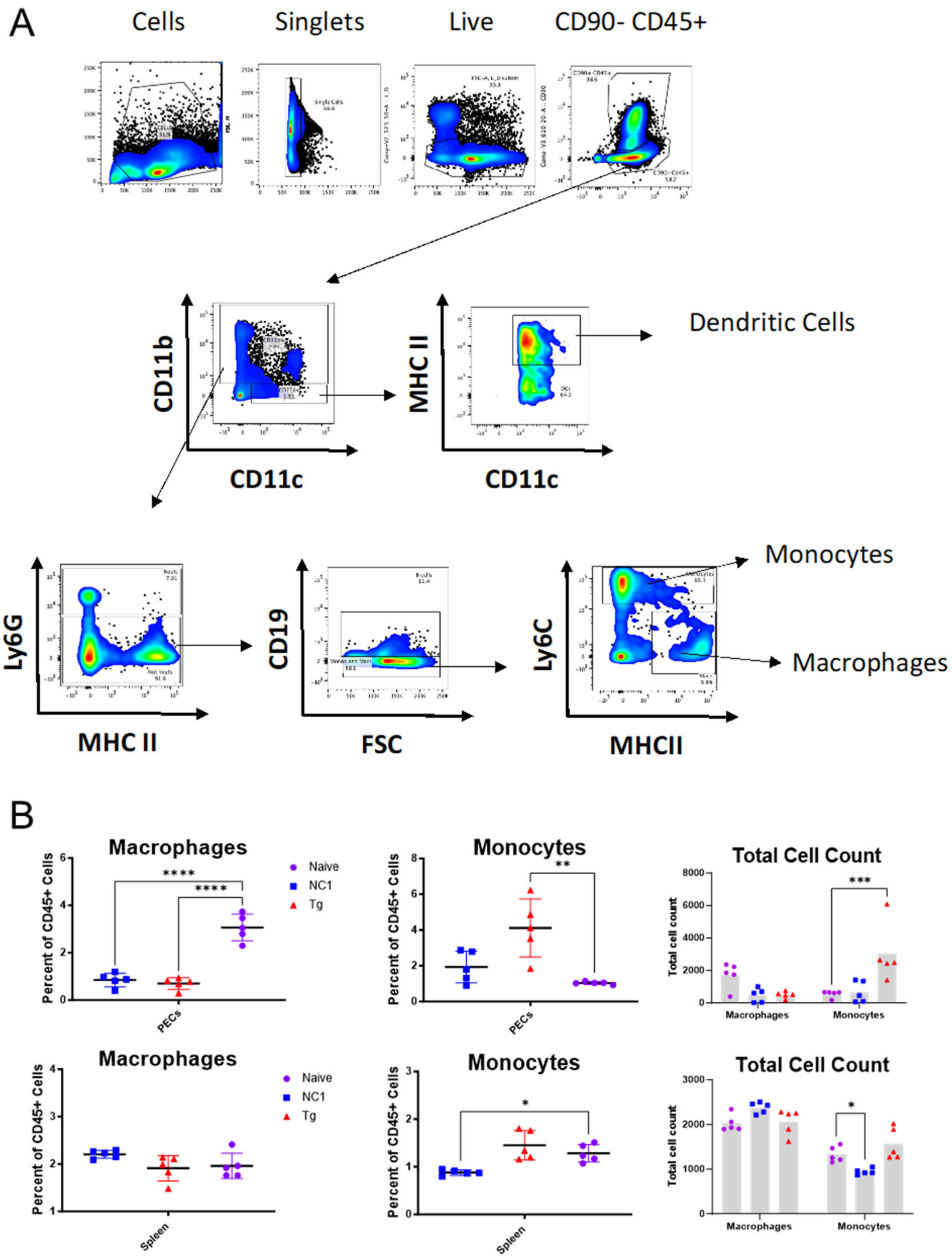

Supplemental Figure 1: A) Gating strategy to identify cell populations in peritoneal and spleen cells. Gating strategy to identify monocyte, dendritic cell and macrophage populations. Clumps and small debris were removed by gating SSC-A x FSC-A. Doublet were removed by gating FSC-H x FSC-W. Live cells were identified using a viability stain (LD) and gated L/D x FSC-A. Leukocytes were then identified based on CD45.2 expression and CD90+ cells were excluded by gating Cd45.2 x CD90. Live CD45+, CD90- cells were gated by CD11b x CD11c to identify

CD11b-, CD11c+ cells and CD11b+ cells. CD11b-, CD11c+ cells were then gated with MHC II to identify CD11b-, CD11c+, MHC II+ dendritic cells. CD11b+ cells were further analyzed by excluding Ly6G+ (neutrophils) and excluding CD19+ (B cells). CD11b+, Ly6G-, CD19- cells were gated by Ly6C^{High} (monocytes) and Ly6C^{Low} MHCII^{High} (Macrophages). B) Quantification of the frequency of macrophage and monocyte populations in spleen and peritoneal cells from infected and naïve mice, and total cell count of each type. Six to eight week old Balb/C mice were infected with *T. gondii* (Tg:S1T,red) or *N. caninum* (Nc:Nc1, blue) and compared with uninfected (naïve, purple) samples. Peritoneal and spleen cells were collected and stained with relevant antibody cocktails. Flow cytometric analysis was used to compare cell populations as in Figure 1. Cell populations are frequencies of live leukocytes (frequency of CD45.2+). Data were analyzed by one-way ANOVA with Dunnett's multiple comparison post-test comparing infected mice to naïve mice. **p*=<0.05, ***p*=<0.01, ****p*=<0.001

Figure S2:

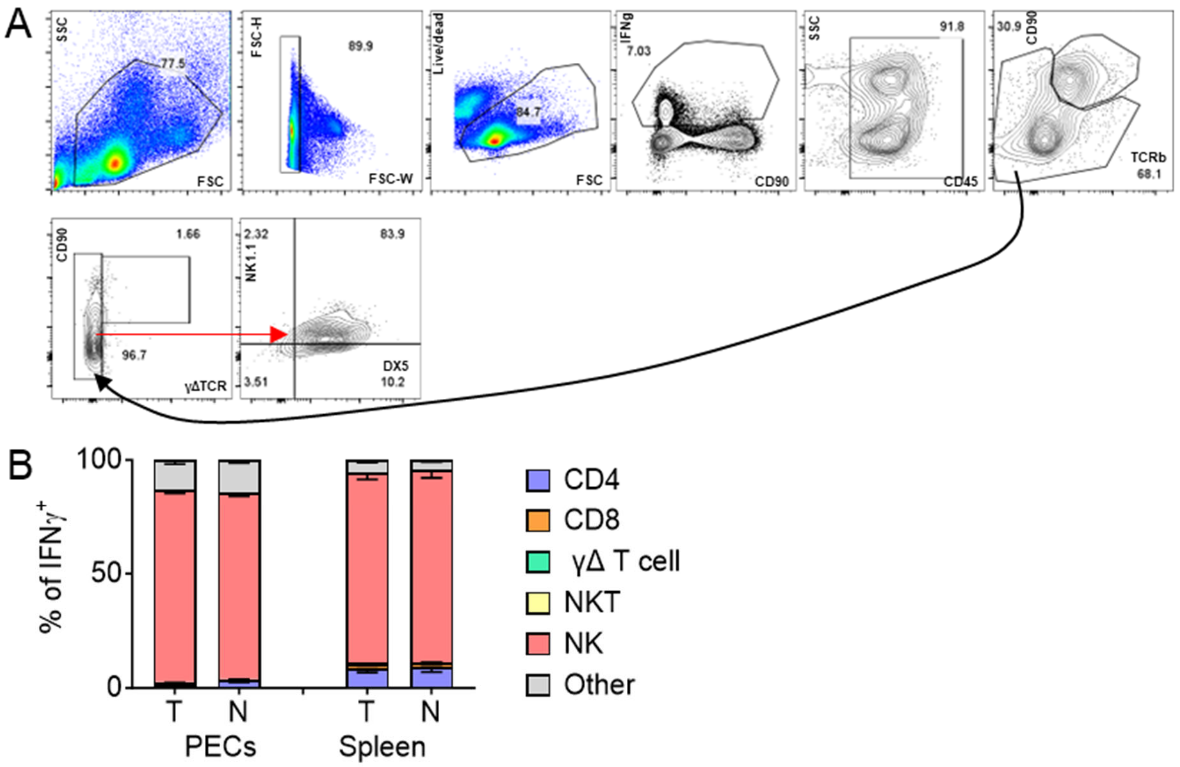

Figure S2: A) Gating strategy to identify IFNγ producing cells in *N. caninum* and *T. gondii*-infected mice. Clumps and small debris were removed by gating SSC-A x FSC-A. Doublets were removed by gating FSC-H x FSC-W. Live cells were identified using a viability stain (LD) and gated LD x FSC-A. Leukocytes were then identified based on CD45.2 expression Live CD45+, CD90- cells were gated by DX5+ (NK cells), while live CD45+, CD90+ cells were gated on γΔTCR for γΔ T cells. NKT cells were DX5+ and CD3+. CD4 and CD8 cells were CD3-positive and CD4 or CD8-positive, respectively. B) Quantification of the frequency of IFNγ+ cells of the indicated cell types in mouse PECs and spleen cells 10 h post-infection with *N. caninum* or *T. gondii*.

Figure S3:

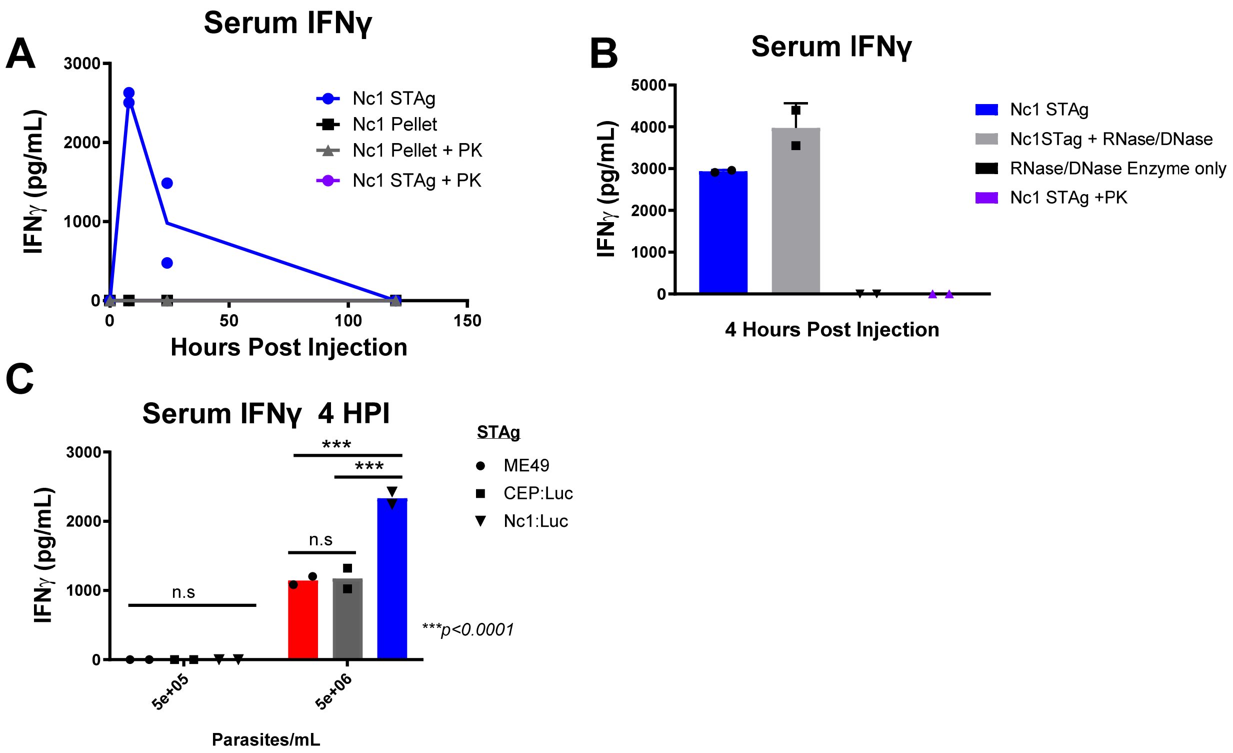

Figure S3: A) Time course of IFNγ induction after injection of mice with *N. caninum* STAg and impact of proteinase K. Only untreated STAg and not proteinase K-treated STAg or the insoluble tachyzoite pellet induced IFNγ in mice. B) Only proteinase K, but not RNAse and DNAse, treatment abrogated IFNγ production by *N. caninum* STAg. C) *N. caninum* STAg induces more IFNγ in mice compared to both *T. gondii* strain ME49 and *T. gondii* strain CEP.

Supplementary Dataset 1: Luminex analysis of 11 cytokines and chemokines from mice infected with *T. gondii* or *N. caninum*. Data are from multiple experiments (which is indicated in the "experiment" column) and with a variety of knockout mouse strains ("genotype" column). Data are extrapolated from a standard curve. "OOR >": out of range of standard curve above; "OOR <": out of range of standard curve below.
